# Catalysing vaccines research, development and manufacturing in Nigeria: a qualitative exploration of industry stakeholders’ knowledge, perceptions and experience

**DOI:** 10.1186/s12961-026-01442-z

**Published:** 2026-03-05

**Authors:** Godspower Onavbavba, Obi Peter Adigwe, Diana Oyin-mieyebi Wilson, Erica Chiamaka Ene

**Affiliations:** National Institute for Pharmaceutical Research and Development, Plot 942, Cadastral Zone C16, Idu Industrial District, Abuja, Federal Capital Territory Nigeria

**Keywords:** Pharmaceutical manufacturing, Public health, Regulation, Collaboration, Vaccine self-sufficiency

## Abstract

**Background:**

The lack of local production of vaccines in Nigeria is a challenge contributing to limited access to healthcare. To address these vulnerabilities, the nation has committed efforts toward vaccine self-sufficiency through local production. However, this initiative has been met with a plethora of constraints. These include inadequate technical capacity, insufficient resources, limited infrastructure, procurement challenges, cold chain management, uncertain market mechanisms and a lack of funding. This study, therefore, aims to bridge this gap by articulating robust strategies for local vaccine production from the perspective of relevant stakeholders across different areas of the vaccine value chain.

**Methods:**

A qualitative strategy was adopted to explore participants’ perspectives on the strategies for sustainable local vaccine manufacturing in Nigeria. The study sample represented stakeholders practising across different areas of the vaccine value chain. Data obtained were analysed thematically to yield themes and sub-themes. Trustworthiness in the study was ensured through recommended frameworks.

**Results:**

Following data analysis, nine overarching themes were identified, which articulated the need to build capacity and an enabling environment for vaccine development in Nigeria. The findings showed that inadequate research capacity, weak infrastructure and poor commercialization frameworks constrain vaccine production, necessitating phased production initiation, strengthened research systems, infrastructural development and improved market access. Effective management and advocacy were found to be critical for securing government and stakeholders’ commitment, as well as reducing vaccine hesitancy to support sustainable local vaccine production.

**Conclusions:**

This study provides in-depth insights into the strategies for expediting the national objective to achieve vaccine self-sufficiency, including the establishment of an enabling policy and legislative environment to attract investment, streamline regulation and secure market access for locally produced vaccines. It emphasizes strengthening research and development capacity beyond fill-and-finish activities, addressing workforce skills gaps through training and technology transfer, and improving infrastructure through reliable power supply and vaccine industrial parks. Overall, the findings highlight the need for sustainable financing, stakeholder coordination, and strong government interventions to ensure the viability of local vaccine manufacturing. These insights can guide effective planning and comprehensive efforts into the requisite areas necessary for sustainable vaccine manufacturing in the country.

**Supplementary Information:**

The online version contains supplementary material available at 10.1186/s12961-026-01442-z.

## Background

Nigeria’s sub-optimal manufacturing in relation to vaccines constitutes a critical challenge in the country. This gap has led the country to rely on the importation of all its vaccines and has resulted in supply chain issues and limited access to this important public health tool [1]. Nigeria expends an estimated US $8 billion annually on vaccine importation, a cost that could be substantially reduced through sustainable local production [2]. From a medicine security standpoint, the lack of local vaccine production in the country not only impedes responses to public health emergencies but also threatens the overall well-being of the population [3]. The impact of this gap was particularly evident during the coronavirus disease-19 (COVID-19) pandemic, when Africa’s vaccination coverage reached only 25% of the population by late 2022, compared with over 60% of global vaccine supplies were secured by developed nations [4]. This disparity underscored the nation’s immense need to enhance its public health system against the vulnerabilities associated with the dependence on imported pharmaceuticals. The importance of attaining vaccine self-sufficiency has, therefore, driven the imperative to build capacity for its local production in the country. Since the COVID-19 pandemic, this disposition has gained prominence and has stimulated efforts by the Federal Government of Nigeria toward the acceleration and improvement of the country’s capabilities in this critical thematic area [5].

In accordance with this, activities geared towards local vaccine production in the last 3 years have given rise to strategic initiatives. These include the development of the Nigeria Vaccine Policy and the attainment of Maturity Level 3 for vaccine licensure in the country [6, 7]. More recently, a National Plan for Vaccine Research & Development and Local Production was launched by the Federal Ministry of Health and Social Welfare (FMoH&SW) in January 2024 [8]. These concerted efforts by stakeholders towards the advancement of local vaccine manufacturing in the country have, however, been met by a plethora of challenges. For instance, vaccine production is a cost-intensive process that demands rigorous adherence to international Good Manufacturing Practice (GMP) and WHO quality standards across research, manufacturing, and distribution [9] which can be hindered by infrastructural limitations, inadequate technical capacity, unstable power supply and the high cost of maintaining validated facilities and robust quality-assurance systems. However, support for meeting these prerequisites remains inadequate in the country, thereby discouraging efforts toward achieving the overarching objective of ensuring medicine security through local production. Manufacturers are identified as having limited infrastructure, inadequate technical capacity or insufficient resources to support the research and development for end-to-end integrated vaccine production [10]. Achieving this requires coordinated investment from government agencies, private-sector partners and international development organizations, focusing on infrastructure, workforce development, technology transfer and sustainable research funding. In addition, the challenges with procuring raw materials, cold chain management and uncertain market mechanisms hinder the justification of investments into vaccine production lines in Nigeria.

As a result, whilst several African countries, such as South Africa, Egypt and Senegal, have attained varying levels of fill-and-finish or full-scale vaccine production capacity, Nigeria remains at a developmental stage [11]. Evidence from literature further reveals that there is a significant dearth of data on the requisite strategies to navigate against the identified country-specific challenges for vaccine production [12]. Although renewed policy efforts have been directed towards vaccine production in Nigeria [6], there still exists minimal engagement in this area, constrained by the paucity of empirical evidence and stakeholder-driven strategies, plans and investments to guide the process of sustainable, end-to-end vaccine manufacturing [13].

Vaccine research and development provide the foundation for manufacturing capacity by enabling antigen identification, process optimisation, and regulatory readiness required for large-scale production [9]. Experience from other low- and middle-income countries demonstrate that sustained research and development investment precedes the establishment of manufacturing capacity. In India and Brazil, government-supported research and technology transfer partnerships enabled a gradual transition from formulation to full vaccine production [13, 14]. In Nigeria, with over 115 pharmaceutical manufacturers [15], human vaccine production has not resumed since the closure of the Federal Vaccine Production Laboratory in 1991 [16]. However, renewed efforts through Innovative Biotech using Virus-like particle platforms, Biovaccines Nigeria Limited employing mRNA tech transfer and partnerships with international manufacturers aim to rebuild this capacity [11]. Research activities relevant to vaccine innovation are conducted within institutions such as the National Institute for Pharmaceutical Research and Development (NIPRD) and the Nigerian Institute of Medical Research (NIMR), with pilot plant facilities supporting early-stage development and clinical trials. However, infrastructural and technical limitations continue to constrain the translation of research and development outcomes into sustainable manufacturing [10, 17].

Recognising these existing complexities, therefore, necessitates the development of comprehensive schemes that will address the identified challenges whilst also initiating the process of local vaccine manufacturing in the country. This will facilitate the responsiveness of the nation to its local context demands and bolster resilience against infectious diseases. The study, therefore, aimed to gain in-depth perspectives from multiple stakeholders across the vaccine value chain on the most appropriate measures necessary for expediting local vaccine production. The articulated strategies can underpin the contextual policy and practice reforms necessary for sustainable local production of vaccines in Nigeria.

## Methods

### Study design

The study employed a qualitative approach and was undertaken to explore the perspectives of relevant stakeholders on the strategies for expediting local vaccine production in Nigeria. Several factors informed the adoption of this framework for the scientific inquiry. Primarily, there was a need to provide an in-depth understanding of the measures required to facilitate local vaccine manufacturing, addressing key challenges such as research and development, infrastructure and distribution, while capturing the full range of barriers across the vaccine value chain. Despite the urgent need for vaccine self-sufficiency in Nigeria, systematic input from experts across the vaccine value chain remains insufficient to effectively inform policy and development strategies. This has, therefore, highlighted the need to generate context-specific insights across all thematic areas relevant to vaccine production, thereby avoiding the limitations associated with a deductive research inquiry. Consequently, an interpretivist approach was therefore employed as this would prove more comprehensive in articulating the strategies to address the intricacies across the vaccine development sector [18]. To best achieve this, the conceptual framework for qualitative research outlined by Creswell [19] was adopted to ensure the generation of findings not arrived at by quantitative methodologies.

### Sampling

Participants in this study were purposefully recruited from the stakeholders who were present at a High-Level Meeting on Local Vaccine Manufacturing convened by the National Institute for Pharmaceutical Research and Development (NIPRD), in the Federal Capital Territory (FCT), Abuja, Nigeria. This meeting was held to generate rich insights into strategies for expediting sustainable vaccine production. The respondents were selected on the basis of their diverse professional backgrounds, years of practice and roles in the value chain. The inclusion criteria for participants in this study were experts with at least 10 years of experience in a dedicated area of the vaccine value chain, who were Nigerians and willing to participate in the research. Respondents were excluded from the study if they had fewer than 10 years of professional experience, were not directly involved in vaccine-related fields such as pharmaceutical manufacturing, regulatory affairs, public health policy or immunisation logistics, or declined to provide informed consent.

### Data collection

The qualitative study utilized a semi-structured interview guide to gain the perspectives of participants. The guide was developed by the authors based on existing literature and study objectives. This approach allowed for flexibility, flow and structure, ensuring that respondents provided in-depth insights on key themes relevant to the research objectives [20]. The interview guide comprised open-ended questions and was structured to obtain socio-demographic details of the participants. Questions focused on the state of vaccine development and strategies for expediting progress in this area (See supplementary file). The guide was pretested amongst three external experts for face and content validity prior to the commencement of data collection to assert its validity in addressing the topic.

To recruit participants, official emails were sent to the identified respondents, informing them of the study objectives and requesting their participation in the research. Upon receipt of consent to participate in the interview and be recorded, sessions with the participants were scheduled to be held physically. Each interview typically lasted for at least 30 min and unfolded such that respondents critically explored issues they felt were essential to facilitating vaccine production in Nigeria. The sessions were documented using both a researcher’s field notes and an audio recorder. The interviews were anonymized, and no person-identifiable data were documented.

At the 15 interview, data saturation was achieved as no new theme was further derived regarding the subject matter. Two interview sessions were subsequently undertaken, which affirmed data saturation, given that emergent themes only buttressed already developed findings [19]. All interviews were conducted in English language, and interview data were appropriately stored and encrypted for confidentiality.

### Ethics consideration

Ethical approval for the study was obtained from the National Institute for Pharmaceutical Research and Development Health Research Ethics Committee. Participation in the study was solicited, and informed consents were thereafter obtained, having conveyed information on the assurance of confidentiality and anonymity to each participant.

### Data analysis

All audio recordings were manually transcribed verbatim by two independent researchers to preserve accuracy and contextual meaning. The data were then analysed thematically using the framework approach [21]. The transcripts were thoroughly read through to gain familiarity with the datasets; thereafter, three levels of coding were undertaken. First, the transcripts were coded line by line to capture the original views of the respondents. This involved the de novo scanning of transcripts to obtain emerging ideas. Thereafter, preliminary codes were assigned to the data. The emergent codes were constantly compared until brief code structures representing diverse concepts were fully defined.

The second level of coding involved the generation of sub-themes by the clustering of code-structures. In addition, textural descriptions to clarify and make sense of the phenomenon were assigned to the sub-themes, having compared and contrasted memo drafts with each generated sub-theme. In the third level coding, the sub-themes were then organized and aggregated on the basis of the semantic content to yield the emergent themes. Each theme was structured to address dominant areas of interest in expediting vaccine production as proposed by the perspectives of stakeholders. The results were presented textually as quotes.

### Quality in data management

Rigour was ensured in this study through the adoption of recommended validation techniques, which were assessed using the criteria of credibility, reliability, transferability and conformability proposed by Lincoln and Guba [22]. Credibility was fulfilled through the analytic triangulation, which entailed the analysis of interviews by two independent researchers. In addition, the verbatim transcripts, field notes, memos, reflective journals and the subjectivity statements of the researchers were constantly referred to throughout the phases of the study to eliminate bias and clarify the researchers’ position.

The transferability and reliability of the study were illustrated using thick descriptions in answering the questions of the Critical Appraisal Skills Programme (CASP) checklist [23]. Also, the confirmability of the study was ensured by an independent lead researcher who thoroughly reviewed the ideas and concepts presented throughout the study. To strengthen confirmability and reliability, an audit trail was ensured throughout the process of investigation.

## Results

### Demography of respondents

The demography of the respondents in this study includes researchers, healthcare practitioners, pharmaceutical manufacturers, regulators, health administrators and policymakers within the vaccine value chain. Of the 17 stakeholders who participated in the study, respondents with a doctoral degree represented a higher frequency of the sample. The cohort practised in varying sectors, with five respondents from the private sector, nine (9) participants from the public sector and others from development agencies. Further details about the demographic characteristics of the sample are presented in Table 1.

**Table 1:** Demographic characteristics of the study participants

Participant number	Gender	Role in the vaccine value chain	Years of practice	Highest level of qualification	Sector of practice
Respondent 1	Male	Manufacturer	40	Master’s degree	Private
Respondent 2	Female	Policy maker	38	Doctoral degree	Public
Respondent 3	Male	Regulator	20	First degree	Public
Respondent 4	Male	Healthcare professional	32	First degree	Development agency
Respondent 5	Female	Health Administrator	23	Master’s degree	Public
Respondent 6	Male	Manufacturer	20	First degree	Private
Respondent 7	Male	Researcher	30	Doctoral degree	Public
Respondent 8	Female	Healthcare professional	35	Doctoral degree	Private
Respondent 9	Male	Researcher	18	Doctoral degree	Public
Respondent 10	Male	Researcher	26	Doctoral degree	Private
Respondent 11	Male	Health administrator	23	Fellow, West African College of Pharmacists (WAPCP)	Public
Respondent 12	Male	Researcher	17	Doctoral degree	Private
Respondent 13	Male	Regulator	20	Master’s degree	Development agency
Respondent 14	Male	Regulator	33	Doctoral degree	Development agency
Respondent 15	Female	Healthcare professional	18	Doctoral degree	Public
Respondent 16	Male	Researcher	26	Doctoral degree	Public
Respondent 17	Male	Researcher	32	Doctoral degree	Public

### Findings

**Fig. 1:**
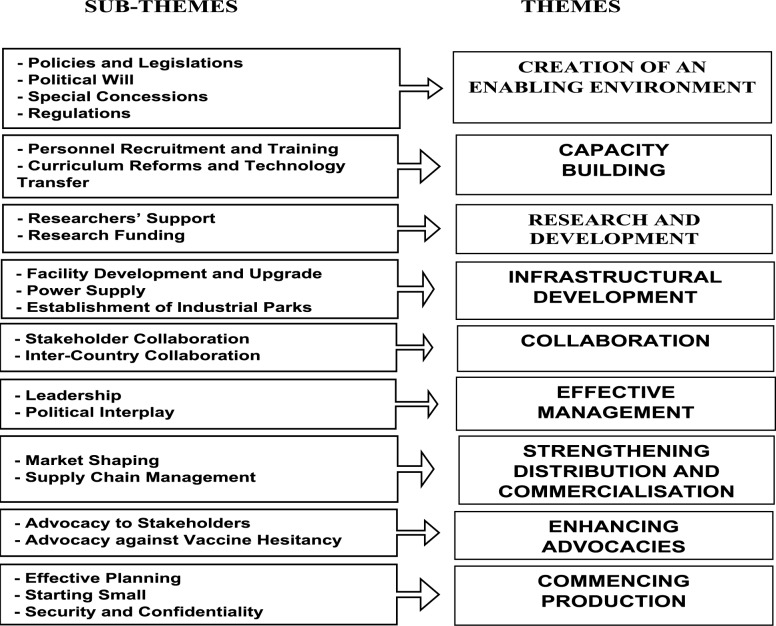
Flow diagram depicting the emergent themes and subthemes

Nine overarching themes on the strategies to expedite local vaccine production in Nigeria were identified from the analysis of the data. The themes include “creation of an enabling environment,” “capacity building,” “research and development,” “infrastructural development,” “collaboration,” “effective management,” “strengthening distribution and commercialisation,” “enhancing advocacies” and commencing production”. Figure 1 provides a summary of the themes and subthemes that emerged from the analysis of data.

1. Creation of an enabling environment

Participants described the creation of an enabling research and manufacturing environment as one of the prior strategies necessary to facilitate vaccine production in the country. This theme buttressed areas relating to policies and legislation, special concessions, regulations and the political-will, wherein the attention of the Government is required to drive the development of vaccines.

- Policies and legislation

To expedite local vaccine production in Nigeria, the respondents reflected on the challenges associated with vaccine access, patronage and overall production, advocating for strong policies in these areas. The stakeholders highlighted that whilst there is an existing vaccine policy in the country, a review of the document to ensure that locally produced vaccines are patronized and accessible to the population was necessary.

*“I know that there are policies, but policies directed towards making sure that production of the vaccines gets to the last man should be there. The National Health Policy needs to be reviewed or updated so that we are able to make sure that we can provide the adequate health needs of our citizens*.” (Respondent 11, male, fellow of WAPCP and 23 years of experience as a health administrator)

“*The Government must do something about making a policy that will create a conducive environment to encourage investments and patronage in the production of vaccines. If there is a law that donor partners coming to us will invest or purchase our local products, then definitely, production of vaccine and commercialization will be very effective*” (Respondent 8, female, with a doctoral degree and 35 years of experience as a healthcare professional)

The responses highlight that establishing a conducive policy environment for vaccine production would attract foreign investment, promote technology transfer by making expertise sharing a required condition for market access, funding, or investment and strengthen other essential enablers of local manufacturing. To achieve this, the participants indicated that there was a need for appropriate policy interpretation, stakeholder mapping and legislation towards vaccine production.

*“The right policies, well interpreted with workable legislation. We should know who should do what and how far-reaching can it be*?” (Respondent 10, male, with a doctoral degree and 26 years experience in research and development).

b. Political-will

The comments of the respondents indicated that to build an enabling environment for vaccine production, it is imperative to ensure that the political willpower to drive reforms in this thematic area exists. This factor recurred throughout the interview and was often described as the main precursor to sustainable vaccine development in the country.

“*The political-will that unilaterally stopped vaccine production in 1991, if that political will is returned and says we want to produce vaccines in Nigeria, all the necessary things that need to be put in place will definitely be*.” (Respondent 15, female, with a doctoral degree and 18 years of experience as a healthcare professional).

“*I think the first step will be for the Government to be able to have that will power to invest. Once the Government takes the initiative to invest in at least one plant, focus on one or two vaccine lines, then others will be encouraged to do so*.” (Respondent 4, male, with a first degree, and 32 years of experience as a healthcare professional).

c. Special concessions

Some of the respondents were of the view that a suitable environment for pharmaceutical manufacturing would entail the provision and accessibility to special concessions such as loans. The finding indicates that emphasis on the political will of the government would be geared towards the reduction of vaccine imports and donations, stimulating an active push from relevant authorities to undertake the production of this important commodity in the country.

*“The Government must make it possible for Nigerian manufacturers to participate by giving them special concessions such as loans, lands, tax incentives... Because most of the things you need, including power, land, and equipment, you cannot do so much without Government support” (Respondent 17, male, with a doctoral degree and 32 years of* experience in research and development).

d. Regulation

The respondents advocated for the relevant bodies to ensure proper regulation and technical support for researchers and manufacturers toward achieving the appropriate standards for vaccine production. This involves building technical skills in vaccine regulation, research and manufacturing through training.

*“Robust and technical quality assurance of international standards has to be there. The regulatory bodies should get set to assist the companies to set up the facilities and provide guidelines for the production of vaccines in Nigeria.”* (Respondent 5, female, with a master’s degree and 23 years of experience as a health administrator).

“*We need a strong regulatory environment guided by strong and effective policies, legislation, or even legal executive orders to help us reduce customs bureaucracies and hasten our registration processes*.” (Respondent 6, male, with a first-degree and 20 years of experience in pharmaceutical manufacturing).

2. Capacity building

As an overarching theme, building capacity for vaccine production encompassed two major strategies, as this is important to facilitate vaccine self-sufficiency in the country.

- Personnel recruitment and training

A recurring phenomenon in this study was the pressing need for the country to build its expertise in the area of vaccine research, development and sterile production. Buttressing this, personnel recruitment and training were highlighted, indicating the need to map out the current gaps in expertise whilst navigating measures to build capacity across those areas.

*“We need workforce. There has to be a potential mapping of the capacities that we have and how that capacity can be scaled up. A lot of manpower should also go into the sector and Universities can actually play a very large part in this. They have a research and development component*” (Respondent 14, male, with a doctoral degree, and 33 years experience as a regulator)

*“We need human capacity development. It is either you bring in scientists who will train your people or send our scientists out to where it is being* done” (Respondent 16, male, with a doctoral degree and 26 years experience in research and development).

b. Curriculum reforms and technology transfer

For capacity building, established contextual protocols for personnel training and skill acquisition were advocated. This sub-theme was two-pronged, as stakeholders were of the perspective that the lack of vaccine development expertise in the country could be addressed by curriculum reforms and technology transfer.

“*If issues on vaccine production are not properly taught maybe in pharmaceutical microbiology, the whole curriculum needs to be properly overhauled or they could have some refresher courses. So that when pharmacists are introduced, they will understand the possibilities and can then go into vaccine production*.” (Respondent 2, female, with a doctoral degree and 38 years of experience in policy making).

“*We may need to start creating curriculum, even in technical schools, to build the capacities of the people to be able to utilize the high-level performance equipment that we are talking about in vaccine production”* (Respondent 13, male with a master’s degree and 20 years of experience as a regulator).

*“The Government needs to formulate profitable partnerships with vaccine production centres in India or any other country where we will be able to benefit from technology transfer with emphasis on the growth of local content*.” (Respondent 1, male with a master’s degree and 40 years of experience in pharmaceutical manufacturing).

3. Research and development

Given the criticality of research and development in a country’s efforts towards vaccine self-sufficiency, participants emphasized the need to strengthen this thematic area. Regarding this, sub-themes in the areas of support to researchers and funding emerged.

- Researchers’ support

The findings from the data analysis revealed that Government support in form of capacity building and funding for researchers can facilitate research and development for vaccines in the country.

*“We need to strengthen research and development. Research and development will give you, including phases on how you will move away from 100 percent importing of vaccines and push it to 30 percent*. *The government should encourage people through funding to go into research on vaccines. These are the things that actually stimulate the country to focus on that area*,” (Respondent 4, male, with a first degree and 32 years of experience as a healthcare professional).

In line with this, the participants emphasized the need to harness the strengths of local vaccine research and development whilst addressing gaps in areas such as clinical trials, the establishment of genetic sequencing banks, and studies on local herbs.

*“We can have a pool of genetic sequences so that by the time we go into vaccine development, it will be easier to have access to these strains that are peculiar to our own environmen*t” (Respondent 9, male, with a doctoral degree and 18 years of practice in research and development).

*“Strengthen clinical trials and capacity building to ensure that if there’s any new development, it is something that we can quickly do the trial within the country, even before we go outside*. (Respondent 4, male, with a first degree and 32 years of experience as a healthcare professional).

b. Research funding

Some of the participants noted that it was necessary to ensure adequate funding for research and development in the country.

*“There’s a need to support with research funding so that it is localized, even if it may not be 100% funded by the Federal Government, there should be opportunities for collaboration with other partners, such that they are able to incentivize vaccine research locally in the country.”* (Respondent 14, male, with a doctoral degree and 33 years of experience as a healthcare professional).

The highlighted strategies for funding include having National budgetary allocations for research and development, as well as loans, partnership funding and grants. From the perspective of a researcher,

“*When government prioritizes vaccines, then they will make adequate budgetary provisions.... The budget has to be within understanding considering the resource scarcity we are facing now.”* (Respondent 7, male, with a doctoral degree and 30 years of experience in research and development).

Respondents opined that there were secondary barriers regarding access to government-allocated funds for vaccine development, which must be addressed, stating that this would ensure seamless access to finance for research and development of these critical public health tools. A manufacturer indicated that,

“T*The Government should remove the secondary barriers to the funding…make it easy for them to access any approved programmes or funding that has been made available*” (Respondent 6, male, with a first-degree and 20 years of experience in pharmaceutical manufacturing).

*“For partnership funds, the pharma industry and research team can work together to combine funds. Or at regional levels, the AU, the WAHO and ECOWAS can come up with a trust fund which could also be part-funded by the AfDB and other global entities, such that countries can be paying little by little*.” (Respondent 14, male, with a doctoral degree and 33 years of experience as a regulator)

4. Infrastructural development

Participants in this study noted the need to develop infrastructure to cater to vaccine development in the country. In line with this, three sub-themes were emphasized.

- Facility development and upgrade

According to the experts, there is a need to upgrade the available facilities across Universities, research institutions and manufacturing entities.

“*We can upgrade what we have to ground, like setting the quality control labs …and then support them with permanent vaccine research infrastructure*” (Respondent 9, male, with a doctoral degree and 18 years of practice in research and development).

“*Set up a standard WHO complaint laboratory so that what we are producing can be exported” * (Respondent 7, male, with a doctoral degree and 30 years of experience in research and development).

b. Power supply

Participants advocated measures to address the constraints associated with erratic power supply in the country, as this factor was indicated to have challenged pharmaceutical manufacturing over the years.

*“Nigeria is a very gas-rich country. Where we site the vaccine plant, we should ensure that we have gas there. That will ensure a stable supply of electricity to maintain this at a cheaper rate*” (Respondent 5, female, with a master’s degree and 23 years of experience as a health administrator).

*“There must be some independent power source to enable the centers to produce.*” (Respondent 2, female, with a doctoral degree and 38 years of experience in policy making).

c. Establishment of industrial parks

For some of the respondents, it was essential to have established industrial parks in the country as this would facilitate vaccine production whilst also reducing manufacturing costs.

*“An industrial park will be very beneficial…where you can have some utilities and research infrastructure equipped in this vaccine manufacturing hub, which can reduce your costs.*” (Respondent 9, male, with a doctoral degree and 18 years of practice in research and development).

5. Collaboration

This overarching theme highlights the need for collaboration between the different stakeholders and entities across the vaccine value chain.

- Stakeholder collaboration

A recurring strategy to expedite vaccine production in this study was the emphasis on stakeholder collaboration, as this would facilitate the distribution of labour and the harmonisation of efforts toward the development of this essential health commodity.

“*Nigeria has a lot of people that are working on vaccines, and one of the things that we need to do is to harness our potential together…coordinate as many of us that have been identified that are into areas of vaccinology, evaluate our strengths and then bring us together. Then we can begin to think ahead*” (Respondent 9, male, with a doctoral degree and 18 years of practice in research and development).

Respondents were of the view that collaboration should be fostered between Universities and research institutions, amongst manufacturers and between researchers and manufacturing entities.

*“We need to collaborate. I would like to see a situation where we’re able to harmonize our researches for better impact*. Also, *it’s not just that narrow, I may decide that I’m not producing vaccines, but I’m going to be either be bringing in vehicles or, vans, cold chain equipment that can help distribution from where it is produced right to where it will be used*.” (Respondent 14, male with a doctoral degree and 33 years experience as a regulator).

Similarly, it was advocated that collaboration should be fostered between the Government and private entities as well as between sectors that may not be directly linked to vaccine production.

“*It covers finance, it covers the Ministry of Budget and Planning, Ministry of Trade and Investment, even the Ministry of Foreign Affairs. We need to carry all of these stakeholders along.”* (Respondent 13, male with a master’s degree and 20 years of experience as a regulator).

*“There should be synergy between the Government and the private stakeholder... because the industries are there to strengthen the Government*.” (Respondent 17, male, with a doctoral degree and 32 years of experience in research and development).

b. Inter-country collaboration

Participants emphasized the importance of engaging other countries as well as international organizations to facilitate vaccine research, development and production in Nigeria.

*“Partnerships with international organisations, we have GAVI as one of such… and also within the continent is key. In that way, we can leverage such competencies so that we can manufacture vaccines in the country and the continent as a whole.”* (Respondent 14, male with a doctoral degree and 33 years of experience as a regulator).

*“The collaboration I am talking about is not within the nation, but between us and others outside. There is more to partnering than just unilaterally doing things on our own. The world is a global village; if you are able to strategise and have a foolproof concept that represents the interests of all, it is a better and more sustainable approach.”* (Respondent 15, female, with a doctoral degree and 18 years of experience as a healthcare professional).

6. Effective management

To achieve collaboration and sustainable vaccine production in the country, emphasis on effective coordination of stakeholders and management of activities was indicated by the respondents. This overarching theme describes management as a critical tool for the attainment of sustainable vaccine development. The participants indicated that this was essential to ensure accountability and sustainability of efforts. Highlighted under this are sub-themes buttressing leadership and political interplay.

- Leadership

As it pertains to effective management, the participants highlighted the importance of commitment from leaders as well as the need to ensure the continuity of vision even with a change in leadership.

*“It takes the commitment of the Government and the stakeholders. The issue of commitment should start with the Federal Ministry of Health and Social Welfare.”* (Respondent 16, male, with a doctoral degree and 26 years of experience in research and development).

*“When there are changes in power from one Government to another, those policies and strategic goals concerning the production of vaccines should never be tampered with... Give all the necessary resources to drive this programme to a statutory agency, a body that is already established by the law, so there is an assurance that even after the end of one political administration, the agency still exists and the mission continues to move on”* (Respondent 11, male, fellow of WAPCP and 23 years experience as a health administrator)

To ensure effective leadership, the participants were of the opinion that artificial intelligence models could be deployed to track efforts and commitments toward projects.

b. Political interplay

Stakeholders further advocated measures relating to political interplay to foster vaccine production in the country.

*“Assuage the Global North, from the onset, if we are able to make it open for them to come and invest and have their interest align with the interest of our own local production, it will guarantee mass production and continuity.* (Respondent 15, female, with a doctoral degree and 18 years of experience as a healthcare professional).

*“Avoid the Government’s interference. The government should have the political will and allow key players to go on without unnecessary interference. Not to bring in people to dictate policies, even when they are not properly endowed to do so*.” (Respondent 2, female, with a doctoral degree and 38 years experience in policy making).

7. Strengthening commercialization

Participants further suggested strategies to address distribution and market concerns associated with vaccines, as gaps in these areas are capable of discouraging efforts toward vaccine production.

- Market shaping

Market shaping was a critical strategy highlighted by the respondents to stimulate vaccine production in the country. Areas relating to pricing, patronage and market access recurred throughout the interview.

“*Market access, anywhere in the world, the Government of that country is involved. It is a matter of giving an advanced procurement order. Government buying in advance becomes very good for the company, the Federal Government, the State Government or the Local Government*” (Respondent 13, male with a master’s degree and 20 years experience as a regulator).

*“Market shaping is very key. Financial institutions and some key players like GAVI and organisations like Clinton Health Access Initiative need to come on board with other international institutions to have a broader discussion on an agreeable funding mechanism and how best our vaccines can be priced.”* (Respondent 14, male with a doctoral degree and 33 years experience as a regulator).

b. Supply chain management

It was advocated that the supply chain management system in the country be strengthened to facilitate efforts towards vaccine security in the country.

*“We need to strengthen our cold chain supply system. There are some of the steps we can take to initiate the process. I also think we have to get the security people involved. If security agencies are involved in the distribution of vaccines across the board, there will be some places where the vaccines will get to”* (Respondent 5, female, with a master’s degree and 23 years experience as a health administrator).

*“The post-marketing surveillance unit in NAFDAC is to ensure that the supply chain is intact. ‘We can leverage AI, a platform that is free to all population. Then you can easily track that this area has not received vaccine*” (Respondent 3, male, with a first degree and 20 years experience in pharmaceutical regulation).

8. Enhancing advocacies

The participants indicated that to encourage sustainable vaccine production in the country, issues relating to poor awareness of the intricacies and benefits of vaccine production, as well as vaccine hesitancy should be addressed through varying advocacy measures.

- Advocacy to stakeholders

This sub-theme addressed challenges associated with poor stakeholder awareness. It encouraged conversations amongst stakeholders in light of the current activities and engagement regarding vaccine development in the country.

*“There is a need to seriously engage the government to emphasize what vaccine production is all about. Encourage them to buy into it and properly equip institutions that are well-endowed to carry out vaccine production. Carry the campaign to other neighbouring countries, West African sub-regions and then Africa at large* (Respondent 8, female, with a doctoral degree and 35 years experience as a healthcare professional).

“*The conversation we’re having now should be escalated to various stakeholders like PMGMAN, pharmacists, physicians, nurses, and what have you.”* (Respondent 2, female, with a doctoral degree and 38 years of experience in policy making).

b. Advocacy against vaccine hesitancy

Participants indicated that vaccine production in the country would be futile if the challenges of vaccine hesitancy are not addressed. Sensitising the public on the importance of vaccines was therefore highlighted as critical to developing vaccine production capacity.

“*First and foremost, we must do a lot of advocacy at the various levels, especially in the rural areas. Intellectuals and people who are well-educated are also hesitant to take vaccines. If you produce vaccine and people are not willing to be vaccinated, then the exercise will be worthless*.” (Respondent 1, male, with a master’s degree and 38 years of experience in pharmaceutical manufacturing).

*“I have advocated that community pharmacies, which are the first point of call for most Nigerians, should be vaccination centres. They can educate the populace and the hesitancy will reduce. Then we can have the demand go higher for vaccines*. (Respondent 2, female, with a doctoral degree and 38 years of experience in policy making).

9. Commencing production

Participants were of the opinion that the best strategy for expediting local vaccine production in the country is to begin production as soon as possible. This overarching theme included four strategic areas of focus.

- Effective planning

The respondents reported that it was important for stakeholders to plan strategically along the scope of research, development, regulation and manufacturing. This finding highlighted the need to map out human, capital and material resources whilst also prioritizing relevant activities to address the current gaps in vaccine development.

*“Let it not be a talk show… we should put up a strategy and put it down in black and white and then put a timeline”* (Respondent 10, male, with a doctoral degree and 26 years of experience in research and development).

*Proper planning needs to be done… Vaccine production takes time. There is a lot of input that needs to be done correctly. There has to be a mapping of the capacities; we need to draw out the whole process involved and then look at how to support. What vaccine do we produce? We need to prioritize, and look at this critically”* (Respondent 11, male, fellow of WAPCP and 23 years experience as a health administrator).

b. Starting small

With regards to commencing production, some of the participants advocated the need to start small whilst looking to scale up production subsequently. In this regard, the findings indicated that it was necessary to start with the production of routine vaccines, thereafter scaling up production to include vaccines for less endemic diseases.

*“You just have to start somewhere. Start small, maybe the regular vaccines for infant immunization like diphtheria vaccines, then scale up to other vaccines that are moderately used, and then to the ones that are not frequently used but are of value.*” (Respondent 3, male, with a first degree and 20 years of experience in pharmaceutical regulation).

The respondents were also of the view that priority should be given to producing vaccines for indigenous use before scaling up production for export purposes.

*“You are developing first for your use, then you start developing for a competitive advantage over others.”* (Respondent 10, male, with a doctoral degree and 26 years of experience in research and development).

On the other hand, it was deliberated to start with the fill-finish processes for vaccine production in the country before progressing to fully integrated vaccine manufacturing with research and development.

*The fill-and-finish process will be for starters because we are just starting.* *To achieve this in a short period of time, the strategy is to go for a fill-finish approach, at least initially, and then go for backward integration until we have become fully integrated.”* (Respondent 8, female, with a doctoral degree and 35 years of experience as a healthcare professional).

*“Build from zero, grow it, and make it to be sustained. Once we have the research and development completed, the finishing will be okay.”* (Respondent 12, male with a doctoral degree and 17 years experience in research and development).

Having indicated various strategies to commence production, participants were of the opinion that vaccine production can be achieved in the country within 2–5 years of commencement.

*“2 years minimum…depending on the type of vaccine we want to start producing initially. The first year should be on research and development. We need about 1 year for that, before we go to mass production. Give us 5 years. For now, we do not have anything on the ground. Unless you run as if it is COVID-19 that is when you can achieve vaccine production in 2 years.”* (Respondent 17, male, with a doctoral degree and 32 years experience in research and development).

*“Performance metrics should be there to track key performance indicators towards reaching your objectives and your goals*.” (Respondent 5, female, with a master’s degree and 23 years of experience as a health administrator).

c. Security and confidentiality

The respondents opined that as research and development of vaccines are being carried out it in the country, it was essential to prioritize security and confidentiality of the process until the expected outcomes have been yielded.

**“Confidentiality from the side of the scientist and the partners working in that industry is very important”** (Respondent 8, female, with a doctoral degree and 35 years of experience as a healthcare professional)

“*The issue of security is very important in the area of research and development, most especially in the vaccine area, so that we do not start incubating some very toxic substance and then allow it to be vandalized by our population. Because of the nature of the vaccine, if you have an accident or any form of error, it will kill a lot of people*.” (Respondent 4, male, with a first degree and 32 years of experience as a healthcare professional).

## Discussion

This study provides robust insights into the strategies required to expedite local vaccine manufacturing in Nigeria. Emergent findings from this study indicate that an enabling policy environment is key to stimulating local vaccine manufacturing in the country. This implies that the objectives to reactivate local vaccine production in the country may not be fully realized if challenges associated with the policy environment are not effectively addressed. A good example is the Human vaccine production, which ceased in 1991 after a government decision to temporarily close the Yaba Federal Vaccine Production Laboratory for upgrading; a process that never occurred due to funding shortages and shifting priorities [16]. Currently, unconducive elements persist, including procurement systems that favour donor-funded imports, limited incentives for local manufacturers and weak mandates for local patronage. The Nigerian vaccine policy of 2021 proposed various strategies, including enhanced incentive structures and an enforceable local patronage mandate [17]. In particular, stakeholders highlighted the need for appropriate policy interpretation to ensure clear and consistent application of existing frameworks in support of local production. They also highlighted stakeholder mapping to systematically identify and assign roles to key actors across government, industry, regulation and research for better coordination. In addition, targeted legislation was stressed to develop workable laws or executive orders that provide incentives, reduce barriers and mandate patronage of local vaccines. Nigeria still faces a diverse set of needs with regard to the manufacturing, research and regulatory environment [3]. Whilst initiatives such as the recently developed National Vaccine Policy, as well as the National Plan for Vaccine Research & Development and Local Production, have been put in place to enable localized production, the findings demonstrate that the strategic implementation of the policy frameworks hinges significantly on coordinated legislative oversight and a strong political will. The review and robust articulation of the existing policies, as well as the strengthening of legislation to provide a conducive environment for local vaccine production, patronage and access, were therefore advocated. This aligns with a study by Ogada [24], which buttressed the pivotal role of government support in driving vaccine production.

To stimulate vaccine development, there is a need to create encouraging incentives and a favourable market environment. Stakeholders emphasized the importance of incentivizing local manufacturing by providing loans, lands and tax holidays, highlighting the socio-economic aspects of sustainable access to vaccines. This is important, as favourable tax policies and legislative frameworks that assure advanced patronage commitments can reduce the cost of production, promote appropriate investments and provide a guaranteed market to investors [25]. These proposed strategies align similarly with the comprehensive measures undertaken by the Chinese Government, where innovation-driven policies have facilitated regulatory advancements, legislative support and increased investment in vaccine development [26, 27]. With the creation of an enabling policy environment across these areas, actualizing vaccine development goals in the nation can be fast-tracked in a similar measure as is achieved in advanced economies. This will expedite the active participation of Nigeria in the global market.

In addition, a robust regulatory system to stimulate activities towards local vaccine manufacturing is crucial. The findings emphasized the need for a technical environment that will enable the regulation and standardization of vaccine research, development and production to internationally acceptable standards. Similar measures have also been advocated across Africa [28]. From the perspectives of the local manufacturers, an effective regulatory environment informed by policies, legislation and executive orders for reduced customs formalities or product registration processes can promote the efficiency of the sector. However, in Nigeria, agencies such as NAFDAC face limited laboratory capacity, staffing shortages and insufficient technical expertise, resulting in delayed product analysis and weak post-market surveillance [29]. These inefficiencies undermine the quality assurance systems, prolong product approvals and erode confidence in locally manufactured medicines, constraining the growth of domestic pharmaceutical production. The regulatory sector, therefore, needs to focus on comprehensively developing the technical expertise to build capacity in this area. The study also identified the need for personnel recruitment and retention across all areas of the vaccine value chain to address the gaps in the requisite skill set and the lack of adequate staff strength. In addition, measures to build the capacity of the personnel, including curriculum reforms, training and technology transfer, were identified. This finding corroborates reports by da Fonseca [14], which elaborated on the need for innovative practices for technology and knowledge transfer to strengthen the local manufacturing of pharmaceuticals in Brazil. In Nigeria, significant progress in this direction has been made, as the country recorded a nomination to receive mRNA technology transfer for vaccine development in 2022 [30]. Whilst the nation looks to leverage technology assistance for local vaccine production, it is also important to reform educational curricula to align with vaccine development and manufacturing.

In terms of research and development, building capacity is critical to promoting innovative research that will address endemic diseases in the country. As earlier established in the background, vaccine research and development provide the scientific and technical foundation upon which manufacturing capacity is built. The findings of this study reaffirm this relationship, as participants consistently described how inadequate research and development infrastructure and limited translational research capacity hinder the transition to full-scale vaccine production. Their perspectives highlight that without domestic capabilities for antigen discovery, process validation and preclinical testing, manufacturing efforts will remain confined to formulation or fill-finish stages dependent on external partnerships. This empirical evidence therefore supports global precedents, such as those of India and Brazil, where sustained research investment and technology transfer initiatives preceded national vaccine manufacturing autonomy [13, 14]. Robust research capacity enables full-scale vaccine manufacturing by supporting antigen discovery, process optimization, preclinical and clinical validation and preparation of regulatory dossiers for GMP-compliant production as seen in India’s Serum Institute, which evolved from research and development foundations to the world’s largest vaccine producer by volume [13]. Leveraging this research commercialization requires sustained funding, strong intellectual property protection and public–private partnerships for scale-up. Unlike technology transfer, which imports established processes through licensing for more rapid deployment, this endogenous approach builds long-term innovation sovereignty but takes more time and investment; thus, technology transfer is easier to implement first, as in Nigeria’s ongoing mRNA partnerships, allowing initial fill-finish before backward integration [31]. From the perspectives of stakeholders, strengthening vaccine research and development provides insights into the genetic sequences and specific microbial strains peculiar to the environment. Vaccine research and development includes discovery such as antigen discovery through bioinformatics and artificial intelligence, sequencing, plasmid and expression vector construction and antibody engineering as well as pre-clinical formulation, animal studies and bioprocess development which collectively underpin modern vaccine innovation. Similarly, this will strengthen manufacturing capacity though this is an approach requiring significant long term investment. To therefore promote research and development, advanced clinical trial facilities, genetic sequencing banks and research funding were advocated as these form the foundational infrastructure for vaccine manufacturing by enabling pathogen characterization, vaccine design, bioprocess development and scale-up and local clinical evaluation aligned with regulatory requirements [9]. Improved financing measures were similarly highlighted as necessary for vaccine development across other African member countries [32]. Strengthening Nigeria’s capacity for sustainable vaccine production, therefore, necessitates expanded financing sources, reduced secondary barriers to accessing funds and improved associated financial regulations as modelled across other sectors in the international space [33]. The study also proposed national budgetary allocations for research and development in addition to loans, partnership funding and grants, as the sector would benefit from such a blended financing model.

Similarly, the major funding gap for infrastructure in the sector [34], prompted the stakeholders’ consensus on the need for infrastructural guarantees towards and manufacturing facility upgrades, stability in power and water supply and improvement of the available supply chain infrastructure. Furthermore, collaborative measures through public–private partnerships for the development of industrial parks were identified as critical to facilitating vaccine production in the country by enabling shared infrastructure, pooled resources and efficient logistics systems that collectively reduce manufacturing costs. This is in accordance with a previous assessment by the Africa CDC [35], and provides a basis for the production of cheaper and cost-effective vaccines to meet the competitive market demands. Adequately meeting the market demands also necessitates collaborative regional and in-country level expertise. The study emphasized multiple stakeholder collaborative engagement to diversify manufacturing competencies and facilitate market shaping. This was similarly highlighted by the reports of the Global Vaccine Alliance [36], and buttresses the need to leverage the opportunities of the expansive domestic market, as well as the converged African market provided by the framework of the African Continental Free Trade Area.

Collaboration amongst stakeholders, however, demands effective oversight and coordination, driven by high-level political leadership to proactively encourage commitment and continuity of efforts. The study revealed that government involvement in vaccine production efforts can facilitate support for manufacturers in terms of meeting international standards and ensuring advanced market commitments for locally produced vaccines. This finding is in consonance with models utilized for the development of vaccine manufacturing capacities in India and China, as jointly assessed by the Africa CDC and the Clinton Health Access Initiative [37], thereby indicating the importance of coordinated Government oversight on vaccine production efforts. Stakeholders, therefore, highlighted the importance of intensifying advocacy for local vaccine production to the government as well as the population. Advocacy to the public regarding locally produced vaccines and efforts geared at strengthening quality assurance and control during manufacturing and strengthening regulatory oversight can reduce hesitancy and provide assurance of sustained demands for local vaccines [38]. However, at present, there is limited public health and market data to guide the willingness of the public to purchase locally-produced vaccines. The study therefore advocated for effective planning models aided by robust data and artificial intelligence-driven ecosystem to monitor supply chain activities as well as stakeholder commitments in these areas. Following this, the need to commence vaccine production at a small scale as a strategic step to build technical expertise, validate production systems and assess market responsiveness before transitioning to large-scale commercial operations. This strategy aligns with Nigeria’s current limited capacity for vaccine production [39], where “starting small” entails initiating lower-capital fill-and-finish operations or routine vaccines at modest volumes, rather than immediate full-scale antigen production. Although vaccine manufacturing is capital-intensive, with economies of scale crucial for reducing per-dose costs, many African countries, including Nigeria, are deliberately using mRNA partnerships to enter the value chain through fill-and-finish operations. This approach reduces initial financial and technical risks while enabling workforce capacity building, strengthening quality systems, demonstrating operational viability and generating early revenue to support a phased transition to upstream antigen production.

The study had some limitations, particularly those inherent in qualitative design and purposive sampling, which may limit the generalizability of the findings to all stakeholders within Nigeria’s vaccine ecosystem. As with most interview-based research, the data relied on participants’ self-reported experience, which could be influenced by recall or social desirability biases. Although the possibility of subjective interpretation cannot be entirely excluded, analytic triangulation and reflective documentation were employed to minimize researcher bias. Furthermore, the inclusion of participants from diverse sectors strengthened the credibility and depth of the insights generated. Consequently, the study offers valuable implications for policy and practice, emphasizing the importance of sustained political commitment, institutional capacity building and multi-stakeholder collaboration to advance vaccine research, development and manufacturing in Nigeria.

## Conclusions

Nine overarching themes emerged from this study, which underpinned critical strategies to guide the sustainable production of vaccines in Nigeria. Need-based areas, including research and development, commercialization, infrastructure and environmental factors, were highlighted, emphasizing the need to prioritize interventions across these settings towards the localisation of vaccine production efforts. Similarly, the findings identified the importance of providing a robust technical environment for standardized regulation of vaccine development and production, as well as the need for adequate financing. Comprehensively addressing the challenges across these sectors through effective planning models aided by a robust data-driven ecosystem can help the country to respond more effectively to vaccine demands, improve supply chain logistics and foster a healthy and sustainable market for vaccines.

Establishing a conducive policy environment through updated legislation, appropriate incentives and mandatory procurement of locally produced vaccines is equally essential for attracting investment, promoting technology transfer and ensuring sustainable market demand. Strong political will, supported by robust regulatory frameworks and special concessions such as loans, tax incentives and land allocation, is critical to creating an enabling ecosystem for vaccine research, development and manufacturing. Capacity building through personnel training, curriculum reforms, technology transfer partnerships and adequate research funding is required to bridge technical and skills gaps across the vaccine value chain. In addition, effective multi-stakeholder collaboration, strong coordination mechanisms, infrastructural upgrades, including stable power supply and the development of industrial parks, as well as deliberate market-shaping strategies are vital for translating research outputs into commercial production. Commencing vaccine production at a smaller scale, such as fill-and-finish for routine vaccines, alongside sustained advocacy to reduce vaccine hesitancy and efforts to strengthen supply chains, will accelerate Nigeria’s pathway towards vaccine self-sufficiency.

These findings collectively emphasize the importance of sustained political commitment, strategic financing, skilled human capital and coordinated multi-stakeholder action to optimize Nigeria’s efforts toward ensuring population access to safe, quality and efficacious vaccines. They provide a foundation for articulating robust policies and legislation for effective production, whilst also guiding activities towards expediting the actualization of the national objectives to achieve medicine security through the localization of research, development and manufacturing. Future research should aim to empirically evaluate the implementation of the identified strategies across different institutional settings, assess their economic feasibility and explore longitudinal policy outcomes over time. Comparative analyses with other African countries that have advanced vaccine production capabilities would also be valuable in identifying transferable models and best practices that could inform the country’s pathway toward sustainable vaccine self-sufficiency.

## Supplementary Information

Below is the link to the electronic supplementary material.

Supplementary material (PDF 21 KB)

## Data Availability

The datasets used and/or analysed during the current study are available from the corresponding author on reasonable request.

## Abbreviations

COVID-19: Coronavirus Disease 2019
FMoH&SW: Federal Ministry of Health and Social Welfare
NAFDAC: National Agency for Food and Drug Administration and Control
WHO: World Health Organization
USP: United States Pharmacopoeia
CASP: Critical Appraisal Skills Programme
WAPCP: West African Postgraduate College of Pharmacists
AU: African Union
WAHO: West African Health Organisation
ECOWAS: Economic Community of West African States
AfDB: African Development Bank
GAVI: Global Vaccine Alliance
PMGMAN: Pharmaceutical Manufacturers Group of Manufacturers Association of Nigeria
mRNA: Messenger Ribonucleic Acid
CDC: Centers for Disease Control and Prevention
CHAI: Clinton Health Access Initiative
