# Supplementary material for: Catalysing vaccines research, development and manufacturing in Nigeria: a qualitative exploration of industry stakeholders’ knowledge, perceptions and experience
Source: Health Res Policy Syst. 2026 Mar 5;24:33. doi: 10.1186/s12961-026-01442-z (PMC13077912; doi:10.1186/s12961-026-01442-z)
Supplement: Supplementary file 1 — Supplementary material (PDF 21 KB) [file 12961_2026_1442_MOESM1_ESM.docx]

**Interview Guide**

**Introduction**

- Introduction of the interviewer, the study, and its objectives
- Briefly mention the place of the participant in the study.
- Assure anonymity and confidentiality.
- Request for permission to use an audio recorder.

**Background Information**

Note the gender and proceed with the following questions.

- How old are you?
- What is your professional background?
- How long have you been practising in your profession?
- What is your highest level of qualification?
- What is your position and role in your place of practice?

**Views on expediting vaccine research and development in Nigeria**

What can you say about vaccine research and development in Nigeria?

**Prompts**

- What is your perception on support by relevant donor partners regarding funding for vaccine R&D activities in Nigeria.
- What are the challenges related to vaccine research and development in Nigeria.
- What do you consider to be the main challenges faced by local vaccine manufacturers in Nigeria?

**Expediting Local Production of Vaccines**

What measures do you feel should be adopted to achieve sustainable local manufacturing of vaccines in Nigeria?

**Prompts**

- What role do policies, regulations, and government initiatives play in vaccine R&D and manufacturing in Nigeria?
- Are there specific policies or regulations that have helped or hindered the progress in this area?
- What could be improved in terms of policy or regulatory frameworks to boost local vaccine production?
- Are there any specific barriers to policy implementation that impede local vaccine R&D?
- What role do international collaborations and partnerships play in supporting local vaccine R&D and manufacturing in Nigeria?
- How can Nigeria strengthen its relationships with international vaccine manufacturers, researchers, and health organizations to promote local vaccine production?

**Closing Remarks**

- - Thank the participant for their time and valuable insights.
  - Ask if there is anything they would like to add that was not covered in the interview.
  - Reaffirm confidentiality and explain what will happen with the data collected.
